# Supplementary figures and images for: A Tenon’s capsule/bulbar conjunctiva interface biomimetic to model fibrosis and local drug delivery
Source: PLoS One. 2020 Nov 3;15(11):e0241569. doi: 10.1371/journal.pone.0241569 (PMC7608904; doi:10.1371/journal.pone.0241569)

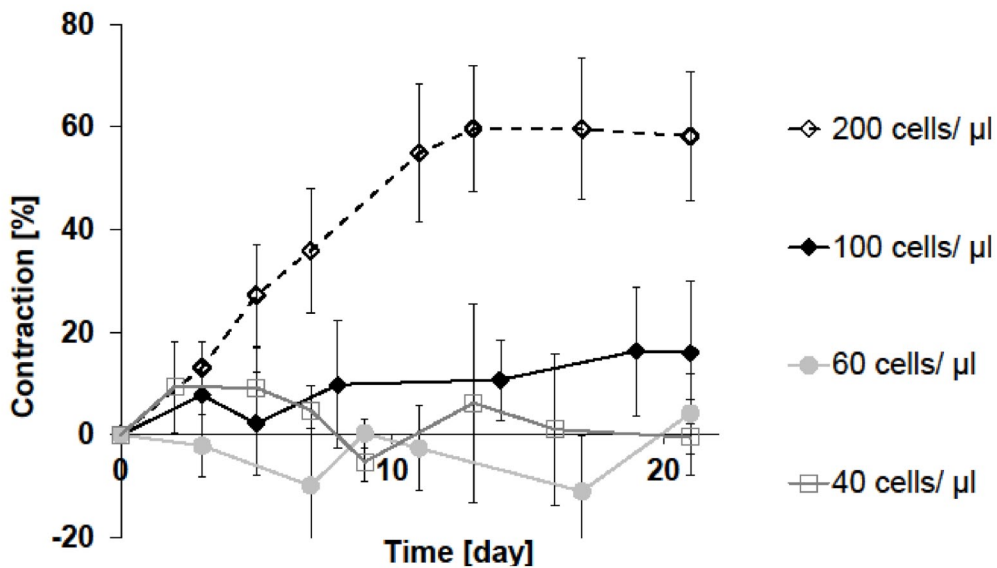

**S1 Fig. Calibration of cell density in compressed hydrogels**

Supplement: S1 Fig — Several cell concentrations of human fibroblasts (HTF9154 cell line) were tested in order to find the most tissue-like contraction pattern. Labels indicate the number of cells per μl of collagen gel before compression. Mean and standard deviation (SD), n = 3–6 gels. (PDF) [file pone.0241569.s001.pdf]

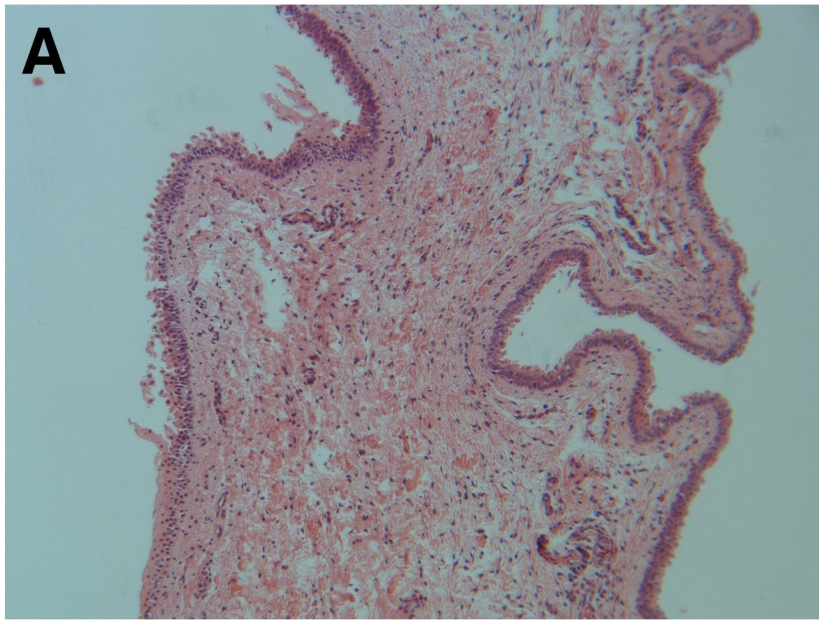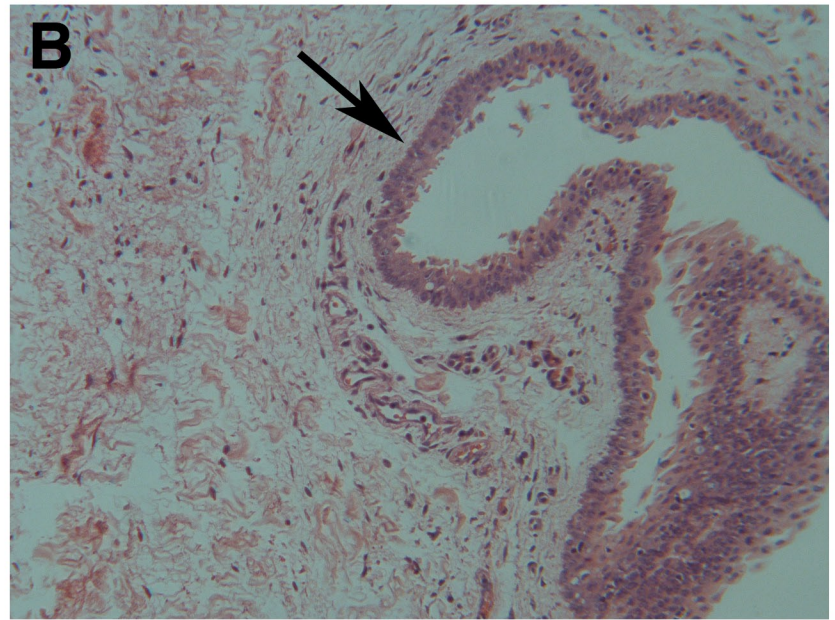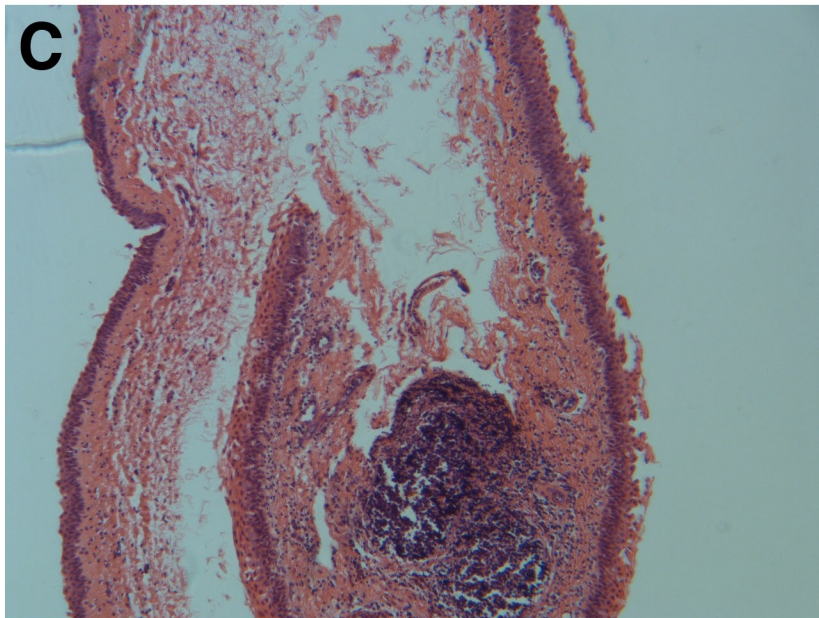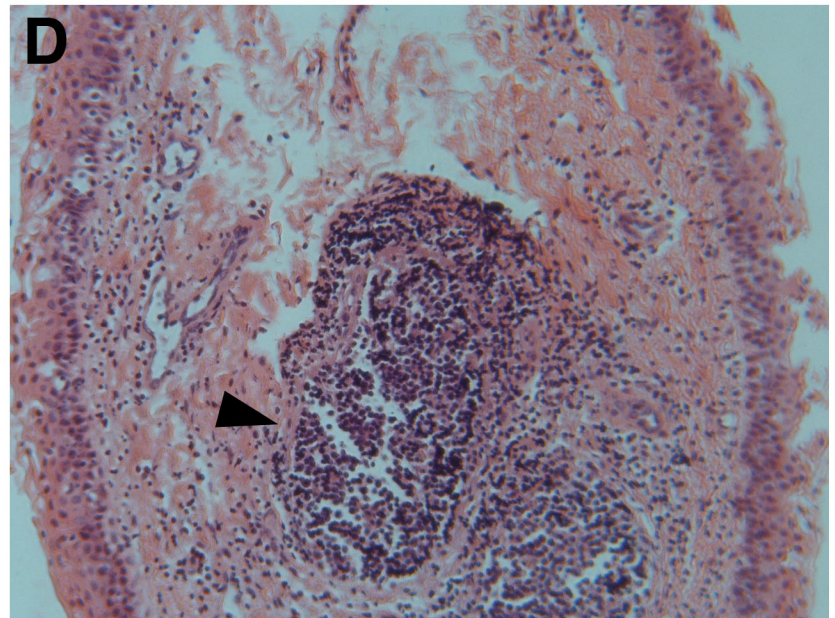

**S2 Fig. Histology of the porcine conjunctiva**

Supplement: S2 Fig — Fresh porcine bulbar conjunctiva fragments representative of those used for the ex-vivo contraction assay were processed for haematoxylin & eosin staining. A, B: porcine bulbar conjunctiva presents a distinct epithelium with about 4 cell layers (arrow). The underlying conjunctival stroma is composed of fibrous matrix populated with immune and stromal cells, which becomes more diffuse and with numerous visible fibroblasts in deeper layers. C, D: immune cell infiltrates are present throughout the conjunctiva, with in some areas discrete conjunctival-associated lymphoid follicles (arrowhead), containing lymphoid cells and macrophages. A, C, 10X magnification; B, D 20X magnification. (PDF) [file pone.0241569.s002.pdf]

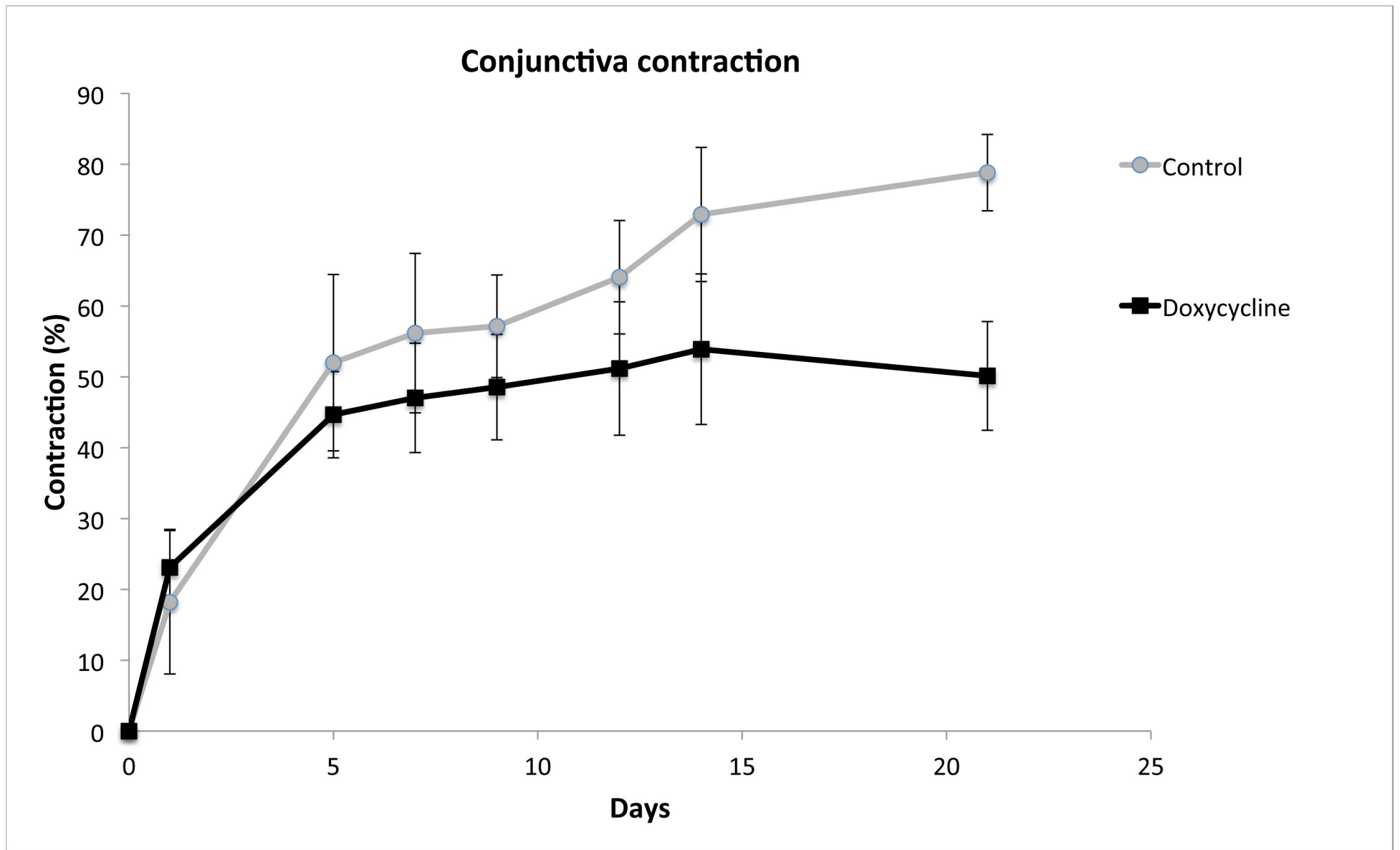

**S3 Fig. Effect of doxycycline on ex-vivo conjunctiva contraction**

Supplement: S3 Fig — Porcine conjunctival fragments were cultured for 2 weeks, with/without 416 uM doxycycline and contraction was measured. Representative experiment, n = 1 (4 fragments each), mean +/- SEM. (PDF) [file pone.0241569.s003.pdf]

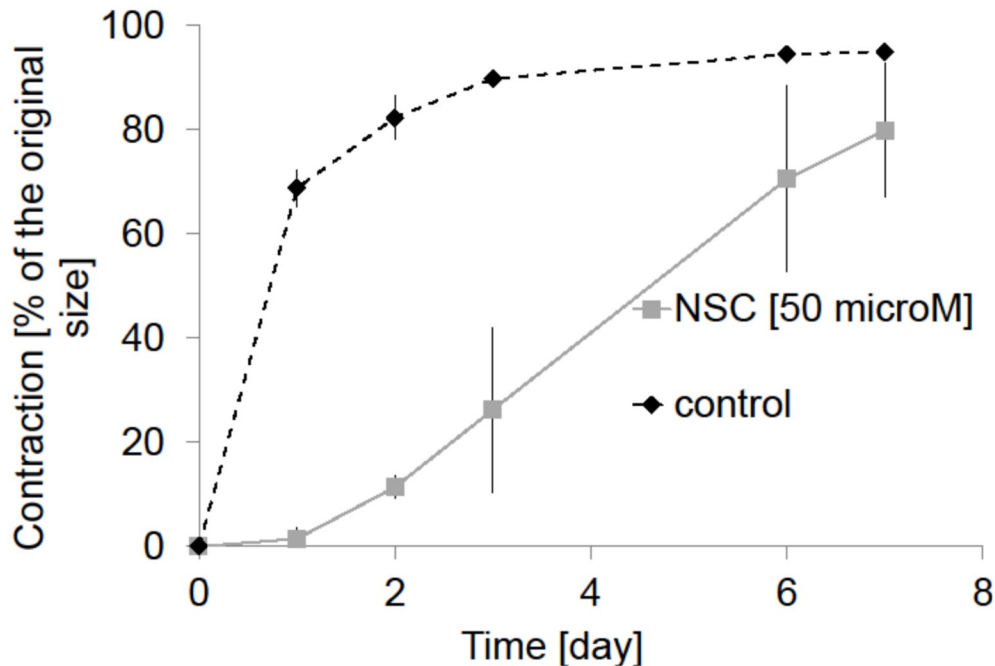

**S4 Fig. Effect of NSC23766 on contraction in non-compressed gels**

Supplement: S4 Fig — NSC23766 treatment leads an initial reduction in contraction in non-compressed collagen gels (standard fibroblast-populated lattices), with the difference decreasing with time. N = 6 gels, one repeat, mean +/- SEM. (PDF) [file pone.0241569.s004.pdf]
